# ABR Features in Ski-Slope Hearing Loss for Hearing Threshold Estimation: A Comparative Clinical Study of Click and CE-Chirp Stimuli

**DOI:** 10.3390/children13030410

**Published:** 2026-03-17

**Authors:** Davide Brotto, Giuseppe Impalà, Elisa Lovato, Elena Mazzaro, Marco Maculan, Elisabetta Zanoletti, Nicole Galoforo, Patrizia Trevisi

**Affiliations:** 1 Section of Otolaryngology, Department of Neurosciences DNS, University of Padova, via Belzoni 160, 35121 Padua, Italymarco.maculan.4@studenti.unipd.it (M.M.);; 2 Unit of Otolaryngology, Azienda Ospedale Università Padova, via Giustiniani 2, 35128 Padua, Italy

**Keywords:** auditory brainstem response, click, CE-Chirp, ski-slope hearing loss, pediatric audiology, high-frequency hearing loss

## Abstract

Background: Auditory brainstem responses (ABRs) are widely used for objective hearing threshold estimation in both adults and children. Click and CE-Chirp stimuli differ substantially in cochlear activation and neural synchrony, yet their relative performance in patients with ski-sloping hearing loss remains insufficiently characterized, particularly with regard to pediatric diagnostic implications. Methods: This study compared ABRs elicited by click and CE-Chirp stimuli in adults with ski-sloping sensorineural hearing loss. The same comparison was also performed in a pediatric cohort including hearing-impaired and normal-hearing children. Adult subjects were further stratified according to audiometric configuration (DROP 1 kHz vs. DROP 2 kHz). ABR thresholds, wave V latency, amplitude, and detectability were analyzed across stimulus types and intensity levels. Associations between ABR thresholds and behavioral audiometric measures were also examined. Results: In adults with ski-sloping hearing loss, CE-Chirp stimulation yielded significantly lower ABR threshold estimates than click stimulation, particularly in the DROP 2 kHz subgroup, and showed stronger correlations with behavioral pure-tone averages across low-, mid-, and high-frequency ranges. Wave V latencies were consistently shorter with CE-Chirp stimulation, while wave V amplitudes did not differ significantly between stimuli at suprathreshold levels. In children, ABR thresholds obtained with CE-Chirp were generally equal to or lower than those obtained with clicks, although statistical significance was limited by sample size. CE-Chirp stimulation was associated with shorter wave V latencies in both hearing-impaired and normal-hearing children and produced larger wave V amplitudes at selected suprathreshold intensities in hearing-impaired children. Conclusions: Click and CE-Chirp stimuli provide complementary information in ABR assessment. While click stimulation remains essential for robust waveform identification, CE-Chirp stimulation appears to offer advantages in threshold estimation and neural synchrony, particularly in ski-sloping hearing loss and pediatric evaluations. Discrepancies between click- and CE-Chirp-derived ABR thresholds should not be attributed solely to maturational or synchrony-related factors but may warrant further frequency-specific audiological assessment to optimize diagnosis and rehabilitation strategies.

## 1. Introduction

Auditory brainstem responses (ABRs) play a central role in objective hearing assessment across the lifespan and are particularly critical in pediatric populations, where reliable behavioral audiometry is often not feasible. ABRs are widely used for newborn hearing screening, diagnostic threshold estimation, and longitudinal monitoring of auditory function. Among the factors influencing ABR outcomes, stimulus type represents a key determinant, as different stimuli engage distinct cochlear regions and neural synchrony mechanisms, in accordance with the tonotopic organization of the cochlea [1].

Click stimuli have long been considered the clinical standard for ABR recordings, providing robust and well-defined waveforms through broadband cochlear activation. However, due to the tonotopic organization of the cochlea and cochlear travel-time delays, click-evoked responses predominantly reflect neural activity arising from the mid-to-high frequency regions, typically between 2 and 4 kHz [2,3,4]. This intracochlear dispersion reduces neural synchrony and may result in decreased response amplitude and less accurate threshold estimation, particularly in specific audiometric configurations [5].

To overcome these limitations, CE-Chirp stimuli were developed to compensate for cochlear dispersion by introducing frequency-specific temporal delays, thereby enhancing neural synchrony across a broader cochlear region [3,6]. The CE-Chirp stimulus, characterized by a flat amplitude spectrum and wide frequency coverage, has been shown to elicit larger response amplitudes and improved waveform morphology compared to click stimuli, especially at low stimulus [7,8,9]. These properties have led to widespread adoption of CE-Chirp stimulation in pediatric audiology and neonatal hearing assessment [10]. However, it is important to note that CE-Chirp stimuli were developed based on latency models derived from normally functioning cochleae, assuming typical frequency-place mapping and normal cochlear tuning [11].

Ski-sloping hearing loss, characterized by relatively preserved low-frequency thresholds and a steep decline at higher frequencies [12,13], represents a particularly challenging condition for ABR-based threshold estimation. In pediatric populations, high-frequency hearing loss may negatively affect speech perception and language development, making early and accurate diagnosis essential [14,15,16]. Importantly, several studies have demonstrated that timely audiological intervention, including early hearing amplification or cochlear implantation when indicated, leads to significant improvements in speech perception and language development in children [17,18]. These findings further emphasize the clinical significance of performing ABR assessments with both click- and CE-Chirp stimuli in a complementary manner to optimize threshold estimation and facilitate early intervention where appropriate. In this context, broadband click stimulation, which predominantly activates basal cochlear regions, may misrepresent auditory thresholds in the presence of steep high-frequency losses [19]. Although CE-Chirp stimulation may provide improved neural synchrony, its diagnostic performance in ski-sloping hearing loss remains incompletely understood, particularly in children.

While previous studies have compared click and CE-Chirp stimuli in normal-hearing individuals and in generalized sensorineural hearing loss [1,11], data focusing specifically on ski-sloping hearing loss are limited. This gap is especially relevant in pediatric clinical practice, where stimulus selection may influence diagnostic interpretation and subsequent rehabilitative decisions.

The present study aims to compare ABRs elicited by click and CE-Chirp stimuli in adults with ski-sloping hearing loss and in a pediatric cohort, with particular attention to threshold estimation, waveform morphology, and stimulus-related discrepancies. The adult cohort provides a reference model in which ABR findings can be interpreted alongside behavioral audiometry, whereas the pediatric cohort represents the primary clinical target, where objective electrophysiological measures are most critically needed. By stratifying adult subjects according to audiometric configuration, this study seeks to provide insights that may inform the interpretation of ABR findings in pediatric settings when ski-sloping hearing loss is suspected.

## 2. Materials and Methods

### 2.1. Ethical Approval Section

This study was conducted in accordance with the principles of the Declaration of Helsinki. Data were examined in compliance with Italian privacy and sensitive data laws and the internal rules of the Padova University Otolaryngology Section. All patients or their parents signed a consent form for privacy disclosure in managing personal data for clinical and scientific purposes.

The present study was approved by the Ethics Committee of the Azienda Ospedale Università Padova (protocol No. AOP2444).

### 2.2. Participants

The study cohort included 32 participants: 24 adults (mean age: 45.7 ± 21.2 years) and 8 children (mean age: 3.1 ± 0.83 months). Adults comprised 14 subjects with ski-slope hearing loss and 10 normal-hearing controls; all patients had a sensorineural ski-slope audiometric configuration. Two subgroups were defined based on the audiometric configuration: (i) subjects with normal thresholds up to 1 kHz followed by a sharp threshold drop at higher frequencies (DROP 1 kHz group) and (ii) subjects with preserved thresholds up to 2 kHz followed by a sharp threshold drop from 4 kHz onward (DROP 2 kHz group).

This stratification (i.e., subdivision in DROP 1 kHz group and DROP 2 kHz group) was based on the hypothesis that the onset frequency of the steep high-frequency decline may differentially influence click- and CE-Chirp-evoked ABR features and threshold estimates due to stimulus-dependent weighting of cochlear contributions.

A control group of normal-hearing subjects was included for waveform characterization analyses. Adult and pediatric cohorts were analysed separately. Normal-hearing adult controls were included for detailed waveform characterization in adults, allowing comparison of ABR morphology and response features with the hearing-impaired adult cohort. Pediatric controls were used exclusively for comparative analyses within the pediatric cohort, due to age-dependent differences in ABR latencies and amplitudes.

The pediatric group included 7 with hearing loss and 7 normal-hearing controls. Participants were rigorously screened to exclude middle ear pathologies or neurological impairments. For pediatric participants, cases with obvious complications of otitis media, syndromic hearing loss associated with structural abnormalities of the middle or inner ear, or other known congenital malformations were explicitly excluded. The term “neurological impairments” was used to refer specifically to clinically diagnosed central nervous system disorders or developmental conditions that could affect auditory processing and ABR reliability, such as cerebral palsy, severe developmental delay, or documented central auditory pathway anomalies. Children with these conditions were not included in the study. These criteria ensured that the pediatric cohort comprised children with isolated sensorineural hearing loss.

### 2.3. Audiometric Assessment

For adults, audiometric evaluations encompassed otoscopy, pure-tone audiometry, speech audiometry, and the Italian Matrix Test (IMT) for speech-in-noise perception. Initially, otoscopy was performed to rule out the presence of impacted earwax or evident pathologies of the tympanic membrane.

Pure-tone audiometry was conducted using the clinical audiometer Madsen Astera2 (Natus Sensory Inc., Schaumburg, IL, USA) in a soundproofed booth. Air conduction thresholds were measured using TDH39 headphones, presenting pure tones at frequencies of 250, 500, 750, 1000, 2000, 3000, 4000, 6000, and 8000 Hz for each ear separately. Bone conduction thresholds were assessed using a bone vibrator placed on the mastoid at frequencies ranging from 500 to 4000 Hz. Contralateral masking was applied when required using narrow-band noise (NBN), calibrated according to Studebaker’s method [20].

To quantitatively describe audiometric configuration, a slope index was derived as a continuous measure reflecting the steepness of the high-frequency threshold decline relative to preserved low-frequency hearing. The slope index was calculated as the difference between average high-frequency and low-frequency pure-tone thresholds, adapting the frequency ranges to the two audiometric subgroups. In the DROP 2 kHz group, the index was computed as the mean threshold at 4 and 8 kHz minus the mean threshold at 0.5, 1, and 2 kHz. In the DROP 1 kHz group, the index was computed as the mean threshold at 2, 4, and 8 kHz minus the mean threshold at 0.5 and 1 kHz. Higher values indicate a steeper ski-slope configuration.

For children, assessments included otoscopy, tympanometry, distortion product otoacoustic emissions (DPOAE), and ABRs to establish hearing thresholds.

### 2.4. ABR Protocol

ABRs were recorded using the Neuro-Audio system Version 1.0.104.1 (Inventis S.r.l., Padua, Italy) using standard surface electrode montage. The nHL values used in this study were based on the calibration provided by the ABR system manufacturer. Click and CE-Chirp stimuli were presented at 80, 60, 40, and 20 dB nHL using alternating polarity. Analysis windows were set at 10 ms for Click stimuli and 15 ms for CE-Chirp stimuli, with a stimulation rate of 21 Hz. Up to 2000 sweeps per stimulus were averaged, with a ±10 mV rejection threshold and a recording noise level (RNL) set at 90 nV as an index of recording quality. Electrode impedance was kept below 3 kΩ and monitored per channel. Signal quality was optimized using adjustable high-pass (0.01–3000 Hz) and low-pass (10–10,000 Hz) filters, with an optional 50/60 Hz notch filter.

For each stimulus and intensity, the presence or absence of wave V was determined, and wave V latency and amplitude were measured when identifiable. The ABR threshold was defined as the lowest stimulus intensity at which a replicable wave V response was identified.

### 2.5. Quality Assessment of ABR Exams

To assess the clinical utility of each stimulus, 10 experienced ABR clinicians were asked to select which trace (click or CE-Chirp) they would use to define the patient’s threshold. ABR recordings from 5 patients were randomly selected, and traces were displayed as images with all identifying information removed to prevent recognition of the stimulus type.

To minimize subjective variability in threshold determination, clinicians apply predefined objective criteria. Specifically, a response was considered present only when waveform components demonstrated high replicability across repeated averages and when peak amplitudes clearly exceeded the estimated residual background noise level (typically ≥3:1 signal-to-noise ratio) [21,22].

### 2.6. Statistical Analysis—Adult Group

Statistical analyses were performed using jamovi (version 2.6.44). Descriptive statistics are reported as mean ± standard deviation or median and interquartile range, as appropriate. Paired comparisons between click- and CE-Chirp-derived ABR thresholds were performed using the non-parametric Wilcoxon signed-rank test, both in the overall hearing-impaired cohort and within audiometric subgroups. The probability of detecting wave V at a given intensity level was analyzed using mixed-effects logistic regression models (binomial distribution, logit link). Stimulus type, intensity, audiometric group, and RNL were included as fixed effects, with subject included as a random intercept to account for repeated measures across intensities and stimuli. Wave V amplitude was analyzed using linear mixed-effects models. Amplitude values were log-transformed to improve distributional properties. Fixed effects included stimulus type, intensity, audiometric group, and RNL, with the subject modeled as a random intercept. Sensitivity analyses were performed by restricting the analysis to suprathreshold levels (40–80 dB nHL) and by testing stimulus-by-intensity interactions. Wave V latency was analyzed using linear mixed-effects models restricted to suprathreshold intensities (60 and 80 dB nHL) to ensure stable waveform identification. Fixed effects included stimulus type, intensity, audiometric group, and slope index, with subject included as a random intercept. Interaction terms were explored in secondary analyses. Given the limited sample size within audiometric subgroups, subgroup-related analyses (including subgroup terms in mixed-effects models and subgroup-stratified comparisons) were considered exploratory and were interpreted cautiously, focusing on effect direction and uncertainty rather than definitive subgroup inference. Associations between ABR thresholds and behavioral audiometric measures were assessed using Spearman’s rank correlation coefficient. Given the ordinal nature of ABR threshold data and the presence of tied values, Kendall’s tau-b was also calculated as a robustness measure.

### 2.7. Statistical Analysis—Pediatric Group

Given the small pediatric sample size, non-parametric statistical tests were adopted, and analyses in this cohort were considered exploratory in nature. Analyses were conducted separately in hearing-impaired children and normal-hearing controls when appropriate. ABR thresholds obtained with click and CE-Chirp stimuli were compared in hearing-impaired children using the Wilcoxon signed-rank test. Differences in wave V detectability at low stimulus intensity (40 dB nHL) were assessed using McNemar’s test for paired binary data. Wave V latency and amplitude were analysed at suprathreshold levels (60 and 80 dB nHL), where responses were consistently identifiable in both groups. Within-group comparisons between click and CE-Chirp stimulation were performed using the Wilcoxon signed-rank test separately in hearing-impaired and control children. To assess whether the magnitude of the click–chirp difference differed between groups, individual difference scores (Δ = click − CE-Chirp) were calculated for latency and amplitude measures, and between-group comparisons were conducted using the Mann–Whitney U test. Wave V amplitude values were log-transformed prior to analysis to reduce skewness and stabilize variance. All analyses of waveform morphology and response features were conducted separately for adult and pediatric cohorts, taking into account differences in age, auditory system maturation, and ABR characteristics.

### 2.8. Statistical Significance

All statistical tests were two-tailed, and a *p*-value < 0.05 was considered statistically significant.

## 3. Results

### 3.1. Audiometric Results of Adult Group

The overall sample included adult subjects with ski-slope hearing loss and a normal-hearing control cohort, analyzed at the ear level (Table 1).

**Figure 1:**
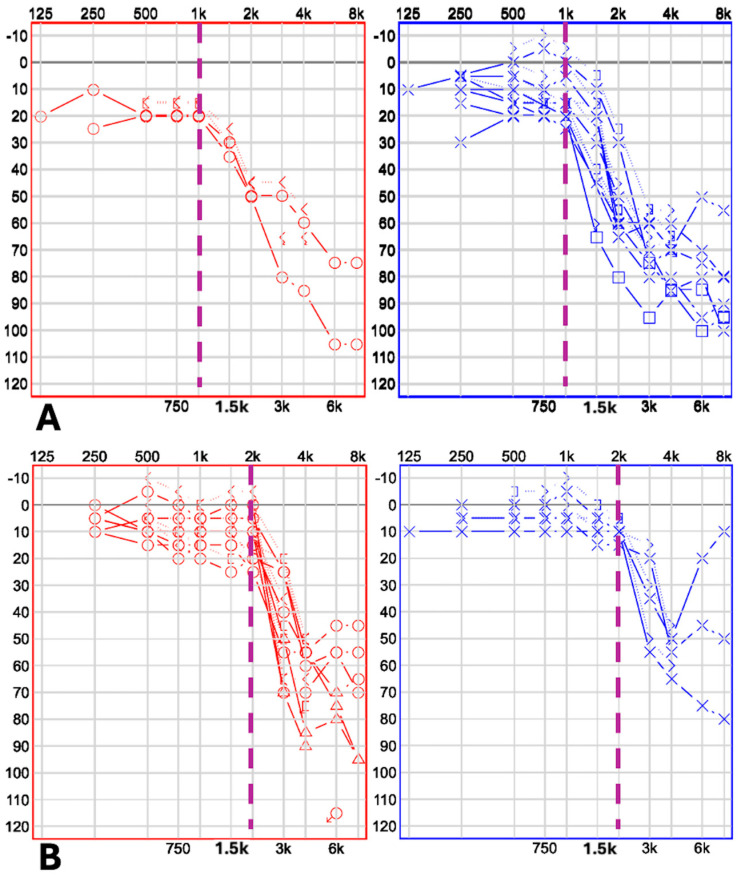
Audiometric threshold configurations of the ears (red for the right ear, blue for the left ear) included in the study, with the panel divided into (**A**) with all DROP 1 kHz thresholds and (**B**) with all DROP 2 kHz thresholds.

The adult ski-slope hearing-loss cohort comprised 21 ears, from 15 males and 6 females, with a mean age of 64.1 ± 12.4 years. Laterality distribution included 10 right ears and 11 left ears. Pure-tone average (PTA), calculated across 500, 1000, 2000, and 4000 Hz, was 31.4 ± 9.9 dB HL. The DROP 1K subgroup included nine ears, from five males and four females, with a mean age of 65.9 ± 17.9 years. Laterality distribution included two right ears and seven left ears. Mean PTA4 was 36.1 ± 10.8 dB HL (see Figure 1A). The DROP 2K subgroup comprised 12 ears, from 10 males and 2 females, with a mean age of 62.8 ± 6.4 years. Laterality distribution included eight right ears and four left ears. Mean PTA4 was 24.6 ± 5.4 dB HL (see Figure 1B).

The normal-hearing control cohort consisted of 25 ears, from 10 males and 15 females, with a mean age of 30.3 ± 15.0 years. Laterality distribution included 12 right ears and 13 left ears. PTA4 values were not reported for this group, as all subjects had hearing thresholds within normal limits and were therefore not directly comparable to the hearing-impaired cohort.

### 3.2. Audiometric Results of Pediatric Group

The pediatric cohort consisted of 14 children, including 7 hearing-impaired (HI) and 7 normal-hearing (NH) participants (Table 1). The mean age at testing for the entire cohort was 3.1 ± 3.5 months (mean ± SD). Nine children were male, and five were female. Testing was performed on seven right ears and seven left ears.

In the hearing-impaired group, the mean age was 4.3 ± 4.8 months, with six males and one female; three right ears and four left ears were tested.

In the normal-hearing control group, the mean age was 2.0 ± 0.8 months, including three males and four females; four right ears and three left ears were tested.

### 3.3. ABR Findings in Adults

ABR threshold estimates obtained with broadband CE-Chirp stimulation were significantly lower than those obtained with click stimulation in the overall hearing-impaired cohort (Wilcoxon signed-rank test, *p* = 0.004). Median ABR threshold decreased from 60 dB nHL with click stimulation to 40 dB nHL with CE-Chirp stimulation (Table 2), with threshold differences occurring in discrete 20 dB steps, reflecting the stimulation protocol. A threshold improvement of at least one 20 dB step was observed in 55.5% of subjects, whereas no difference was found in 38.9%, and a worse threshold with CE-Chirp was observed in a single case. Subgroup analysis revealed that the threshold advantage of CE-Chirp was particularly evident in subjects with hearing loss onset at 2 kHz (DROP 2 kHz group; *p* = 0.006), while no statistically significant difference was observed in subjects with earlier onset of hearing loss (DROP 1 kHz group; *p* = 0.293).

Mixed-effects logistic regression modeling of wave V detectability showed no significant effect of stimulus type or recording noise level (RNL) on the probability of identifying wave V at a given intensity, indicating comparable detection probabilities for click and CE-Chirp stimuli. In contrast, audiometric groups significantly influenced wave V detectability.

Data about wave V amplitude are reported in Table 3. Wave V amplitude analysis using linear mixed-effects models demonstrated a significant effect of stimulus intensity, with larger amplitudes observed at higher intensities, while increased RNL was associated with reduced amplitudes. No significant main effect of stimulus type was observed. Sensitivity analyses restricted to suprathreshold intensities (40–80 dB nHL) and including stimulus-by-intensity interaction terms did not reveal any differential amplitude effects between click and CE-Chirp stimulation.

Data about wave V latency are reported in Table 3. Wave V latency analysis at suprathreshold intensities (60 and 80 dB nHL) revealed a robust main effect of stimulus type, with significantly shorter latencies for CE-Chirp compared to click stimulation (mean difference ≈ 1.5 ms, *p* < 0.001). As expected, higher stimulus intensities were associated with shorter latencies. Audiometric group and slope index did not show significant main effects; however, exploratory interaction analyses suggested that stimulus-related latency differences varied with intensity and audiometric configuration.

Correlation analyses between ABR thresholds and behavioral audiometric measures showed that both click- and CE-Chirp-derived thresholds correlated with high-frequency PTA (2–4 kHz), with stronger associations observed for CE-Chirp thresholds, as indicated by both Spearman’s rho and Kendall’s tau-b coefficients. Additionally, CE-Chirp thresholds were significantly correlated with low- (0.5–1 kHz) and middle-frequency (1–2 kHz) PTA values, whereas click thresholds were not.

### 3.4. ABR Findings in Children

In hearing-impaired children, ABR thresholds obtained with CE-Chirp stimulation were generally equal to or lower than those obtained with click stimulation. Paired comparisons showed a trend toward lower thresholds with CE-Chirp, although statistical significance was not reached due to the limited sample size.

Wave V detectability at low stimulus intensity (40 dB nHL) was further explored using paired binary analysis. At this intensity level, wave V was observed exclusively with CE-Chirp stimulation in one child, whereas no cases showed wave V exclusively with click stimulation. In the remaining children, wave V was either present or absent with both stimuli. The McNemar test did not reveal a statistically significant difference (*p* = 0.317), although the pattern of discordant responses consistently favored CE-Chirp stimulation (Table 3).

Wave V latency was analyzed at suprathreshold intensities (60 and 80 dB nHL), where responses were consistently identifiable in both hearing-impaired and normal-hearing children. In hearing-impaired children, wave V latency was significantly shorter with CE-Chirp stimulation at both 80 dB nHL (Wilcoxon signed-rank test, *p* = 0.016) and 60 dB nHL (*p* = 0.031). Similarly, in normal-hearing children, CE-Chirp stimulation resulted in significantly shorter wave V latency at 80 dB nHL (*p* = 0.008), while at 60 dB nHL a comparable trend was observed without reaching statistical significance (*p* = 0.055).

Between-group comparisons of click–chirp latency difference scores (Δ latency = click − CE-Chirp) revealed no significant differences between hearing-impaired and control children at either intensity level (80 dB: *p* = 0.318; 60 dB: *p* = 1.000), indicating that the magnitude of the latency reduction associated with CE-Chirp stimulation was comparable across groups.

Wave V amplitude analyses were performed on log-transformed values to account for distributional skewness. In hearing-impaired children, CE-Chirp stimulation produced significantly larger wave V amplitudes than click stimulation at 60 dB nHL (Wilcoxon signed-rank test, *p* = 0.016), whereas no significant difference was observed at 80 dB nHL (*p* = 0.172). In contrast, no significant amplitude differences between CE-Chirp and click stimulation were observed in normal-hearing children at either intensity of 80 dB nHL (*p* = 0.498) or 60 dB nHL (*p* = 1.000).

Between-group comparisons of click–chirp amplitude difference scores did not reveal statistically significant differences at either intensity level (80 dB: *p* = 0.181; 60 dB: *p* = 0.073), although a trend toward larger amplitude differences at 60 dB nHL was observed in the hearing-impaired group.

### 3.5. Quality Assessment Results of ABR Exams

Across the 50 evaluations (5 traces evaluated by each of 10 clinicians), click-evoked traces were selected as the most suitable for a threshold identification in 45 cases (90%).

## 4. Discussion

The present study examines the behavior of click and CE-Chirp stimuli in ABR recordings in the specific context of ski-sloping hearing loss, with the aim of improving hearing threshold estimation and informing pediatric diagnostic interpretation. By combining threshold-level and suprathreshold analyses and stratifying subjects according to audiometric configuration, the study provides a framework for understanding how stimulus-dependent ABR features interact with cochlear frequency-specific dysfunction.

Broadband stimuli such as clicks and CE-Chirps do not provide frequency-specific threshold information but rather reflect the functional contribution of the most responsive cochlear regions. The aim of the present study is therefore to compare how these commonly used broadband stimuli behave in the presence of a ski-sloping audiometric configuration.

In adults with ski-sloping hearing loss, CE-Chirp stimulation resulted in lower ABR threshold estimates compared with click stimulation in a substantial proportion of cases, particularly in subjects with preserved hearing up to 2 kHz (DROP 2 kHz group). This finding aligns with the stronger correlations observed between CE-Chirp-derived thresholds and behavioral PTA values across low-, mid-, and high-frequency ranges.

In contrast, click-derived thresholds were only associated with high-frequency PTA. These results suggest that CE-Chirp stimulation may provide a more global representation of cochlear function when mid-frequency regions are still functional. This observation is consistent with the physiological principles underlying chirp stimuli. CE-Chirp was specifically designed to compensate for cochlear travel-time delays by temporally aligning frequency components, thereby enhancing neural synchrony across a broader cochlear region [3,4,6]. Conversely, although click stimuli are broadband, their ABRs predominantly reflect activity originating from the basal cochlea, typically corresponding to the 2–4 kHz region [2].

In the presence of a steep high-frequency hearing loss, this basal bias may lead to ABR threshold estimates that overrepresent high-frequency dysfunction and underestimate residual low- and mid-frequency hearing, as previously described in sloping audiometric configurations [19].

Importantly, the advantage of CE-Chirp stimulation was not observed uniformly across all audiometric profiles. In subjects with hearing loss onset at 1 kHz (DROP 1 kHz group), no significant threshold differences were found between stimuli. This suggests that when cochlear dysfunction extends into lower-frequency regions, the benefits of temporal compensation provided by CE-Chirp may be reduced. This finding supports previous observations that chirp efficacy depends not only on hearing loss severity but also on the frequency range of preserved cochlear function [11]. Such observations are further supported by recent evidence reported by Belet et al. (2025), who compared LS CE-Chirp and click stimuli in subjects with high-frequency hearing loss and similarly observed that chirp-based responses provide advantages that are modulated by the distribution of residual cochlear function rather than by hearing loss severity alone [23]. Their results support the view that stimulus-dependent ABR differences should be interpreted in relation to audiometric configuration, reinforcing the physiological basis and clinical relevance of our observations. This emphasizes the importance of audiometric configuration in ABR interpretation.

At suprathreshold intensities, CE-Chirp stimulation consistently produced shorter wave V latencies than click stimulation (1.5 ms as reported in previous reports), a finding that closely mirrors previous reports in both normal-hearing and hearing-impaired populations [7,24]. This latency reduction reflects enhanced neural synchrony and confirms that the chirp stimulus effectively aligns cochlear responses even in the presence of altered cochlear mechanics. However, no significant amplitude advantage for CE-Chirp stimulation was observed at suprathreshold levels in adults. This pattern is consistent with prior evidence indicating that, at higher intensities, chirp stimuli may induce spread of excitation upward, leading to partial neural desynchronization and attenuation of amplitude benefits [25,26].

This intensity-dependent behavior supports the notion that CE-Chirp stimulation is optimally suited for near-threshold recordings, whereas click stimulation retains advantages in waveform robustness at higher stimulation levels.

The pediatric results, although limited by sample size, followed trends comparable to those observed in adults and were largely consistent with existing pediatric literature. In hearing-impaired children, CE-Chirp-derived thresholds were generally equal to or lower than click-derived thresholds, and wave V latencies were significantly shorter with CE-Chirp stimulation in both hearing-impaired and normal-hearing children. These findings agree with previous studies demonstrating improved neural synchrony, larger amplitudes, and reduced test time with chirp stimuli in neonates and infants [9,27,28].

The observed pediatric latency and amplitude patterns should also be interpreted considering auditory system maturation. ABR wave latencies are known to be prolonged in early infancy, particularly at higher frequencies, and wave amplitudes show age-dependent growth during the first years of life [29,30]. In this developmental context, CE-Chirp stimulation may facilitate wave V identification at lower intensities by recruiting a broader frequency range and optimizing neural synchrony, thereby supporting more reliable threshold estimation when behavioral measures are unavailable. Since behavioral audiometry cannot be used as a reliable comparative benchmark in infants, the adult cohort provides an important interpretative framework for contextualizing the stimulus-dependent ABR patterns observed in the pediatric recordings. However, when considering the relative differences between click and CE-Chirp responses within the same subject, the observed stimulus-dependent patterns cannot be fully explained by maturational factors alone. Given that steep high-frequency losses alter basilar membrane excitation patterns and prolong wave V latency in click-evoked responses, discrepancies between click and CE-Chirp-derived ABR measures in children should be interpreted with caution and may reflect underlying audiometric configuration rather than maturational effects alone.

Despite these physiological advantages, the clinical usability of the two stimuli differed. In blinded trace evaluations, experienced clinicians overwhelmingly preferred click-evoked waveforms for threshold identification. This finding highlights a critical and often underappreciated aspect of ABR diagnostics: waveform interpretability and examiner confidence remain central to clinical decision-making. In this context, it is important to note that recent advancements aimed at improving acquisition efficiency, such as parallel ABR (pABR), primarily address recording time rather than the underlying physiological interaction between stimulus type and cochlear activation patterns. Therefore, understanding how different stimuli behave in specific audiometric configurations remains essential for accurate interpretation, regardless of recording speed. While CE-Chirp stimulation may offer closer correspondence with behavioral thresholds, click stimulation continues to provide sharper waveform morphology and greater visual clarity, particularly in complex audiometric configurations such as ski-sloping hearing loss.

Despite the value of including a relatively understudied population of patients with ski-sloping hearing loss, this study has several limitations. First, the pediatric cohort was relatively small, reflecting the practical constraints associated with extended ABR testing protocols in early infancy. Consequently, analyses in the pediatric cohort should be regarded as hypothesis-generating rather than confirmatory. A further limitation of the study is that adult-onset ski-sloping hearing loss may differ qualitatively from pediatric-onset hearing loss in terms of underlying pathophysiology. Age-related changes and cumulative environmental exposures likely affect the audiometric configuration in adults, limiting the direct physiological comparability of adult ABR data to pediatric responses. Second, in children, a complete frequency-specific audiological profile is not yet available due to age-related constraints of our cohort (but we chose a cohort that could be realistic for the everyday ABR clinical use). Third, a subset of adult cases could not be included in the statistical analyses because of missing data (thus limiting the cohort of adults). In addition, stratification of the adult hearing-impaired cohort into DROP 1 kHz and DROP 2 kHz subgroups resulted in relatively small subgroup sizes. Subgroup-related findings should therefore be interpreted cautiously and considered exploratory. Fourth, only CE-Chirp stimuli were evaluated, and LS-Chirp stimuli were not tested (and this might have revealed even additional data). Fifth, ABR threshold estimation was performed using 20 dB step sizes rather than finer intensity increments (e.g., 5–10 dB), which may have reduced threshold resolution. Therefore, the results should be interpreted in terms of relative differences between stimuli rather than precise threshold estimation. Finally, our protocol did not include the use of frequency-specific stimuli such as tone pips or tone bursts. While broadband chirps are highly effective for maximizing neural synchrony and identifying a global electrophysiological threshold, the absence of frequency-specific testing limits our ability to provide a detailed mapping of the audiometric configuration across discrete frequencies. In cases of sloping hearing loss, the ABR threshold obtained with broadband stimuli may primarily reflect the hearing sensitivity of the most preserved cochlear regions, potentially leading to an underestimation of hearing loss at poorer-frequency regions.

Moreover, the inclusion of both adult and pediatric cohorts introduces inherent methodological constraints. Adult ABR recordings were used as a reference model in a well-defined audiometric condition, in the absence of comparable behavioral thresholds in the pediatric cohort, but they cannot be considered a direct physiological proxy for pediatric responses due to known differences in auditory system maturation and recording optimization strategies. Therefore, cross-population interpretations should be made with caution.

From a clinical standpoint, discrepancies between click- and CE-Chirp-derived ABR thresholds in children should therefore not be interpreted solely as maturational effects or differences in neural synchrony. Rather, such discrepancies may represent a potential indicator of an underlying ski-sloping hearing loss. In the absence of alternative clinical explanations, these findings should prompt further frequency-specific evaluation, including tone-burst ABR or auditory steady-state response (ASSR), with particular attention to the mid-to-high frequency range.

Overall, the present study supports a complementary use of click and CE-Chirp stimuli in ABR assessment. Click stimulation remains indispensable for reliable waveform identification and clinical confidence (i.e., otoneurologic evaluation of the neural auditory pathway), whereas CE-Chirp stimulation provides advantages in threshold estimation and neural synchrony, particularly near threshold (i.e., in ski-slope patients) and in pediatric settings (i.e., in preterm infants for improved threshold definition). An integrated, stimulus-specific approach may therefore improve diagnostic accuracy and support more informed rehabilitative planning in children with suspected high-frequency hearing loss. The discrepancy between click- and CE-Chirp-derived ABR thresholds may also represent a potentially useful clinical cue for non-flat audiometric configurations, warranting further investigation with frequency-specific measures.

## 5. Conclusions

This study highlights the complementary value of click and CE-Chirp stimuli in ABR-based hearing threshold estimation, especially for ski-sloping hearing loss.

Click stimulation remains a robust and reliable reference, providing clear and highly interpretable waveforms across a wide range of intensities, even in complex audiometric profiles. CE-Chirp stimulation, by enhancing neural synchrony and engaging broader cochlear regions, may provide threshold estimates closer to overall hearing sensitivity. This is particularly relevant near threshold and in pediatric assessments, where low-intensity stimuli help minimize discomfort and ensure reliability in auditory threshold determination.

However, acknowledging the sample size and the complexity of cochlear hearing loss configurations, these results should be interpreted as a validation of the clinical methodology and a hypothesis-generating framework rather than definitive evidence of stimulus specificity.

These findings support the integration of both stimuli into ABR protocols. A combined approach may improve diagnostic accuracy across diverse patient populations, addressing the specific needs of both adult and pediatric assessments, especially for high-frequency hearing loss. Future studies involving larger pediatric cohorts and frequency-specific outcome measures are warranted to further refine ABR protocols and optimize clinical decision-making. In addition, future research should explore the potential advantages of Level-Specific (LS) CE-Chirp stimuli, which adapt temporal compensation according to stimulation level. Comparative investigations between standard and LS-Chirp paradigms may therefore help clarify the role of stimulus design in configuration-dependent ABR interpretation.

## Figures and Tables

**Table 1:** Demographic and audiometric features of the studied cohorts.

Cohort	Group	N (Ears)	Age (Mean ± SD)	Male/Female	Right/Left	PTA4 (dB HL)
Adults	Ski-slope HL (overall)	21	64.14 ± 12.39 years	15/6	10/11	31.43 ± 9.86
Adults	DROP 1 kHz	9	69.56 ± 10.64 years	5/4	3/6	41.25 ± 5.04
Adults	DROP 2 kHz	12	60.08 ± 12.45 years	10/2	7/5	24.06 ± 4.50
Adults	Normal-hearing controls	25	30.28 ± 14.98 years	10/15	12/13	6.88 ± 4.98
Children	Hearing-impaired	7	4.29 ± 4.79 months	6/1	3/4	Not available
Children	Normal-hearing controls	7	2.00 ± 0.82 months	3/4	4/3	Not available

Abbreviations: HL, hearing loss; PTA4, pure-tone average across 0.5, 1, 2, and 4 kHz (dB HL); SD, standard deviation.

**Table 2:** Data analysis of the cohorts in terms of wave V comparison in terms of threshold and statistical significance.

Group	N (Ears)	Threshold Click, Median (IQR) (dB nHL)	Threshold CE-Chirp, Median (IQR) (dB nHL)	*p*	Wave V Present 40 dB (Click), n/N (%)	Wave V Present 40 dB (CE-Chirp), n/N (%)	*p* (McNemer)
Adults—Ski-slope HL (overall)	21	60 (60–80)	40 (40–60)	<0.001	4/21 (19.0%)	13/21 (61.9%)	0.003
Adults—DROP 1 kHz	9	80 (60–80)	60 (50–80)	0.021	0/9 (0.0%)	2/9 (22.2%)	0.157
Adults—DROP 2 kHz	12	60 (40–60)	40 (40–40)	<0.001	4/12 (33.3%)	11/12 (91.7%)	0.008
Children—Hearing-impaired	7	60 (60–60)	60 (50–60)	0.157	1/7 (14.3%)	2/7 (28.6%)	0.317

Abbreviations: HL, hearing loss; IQR, interquartile range; nHL, normalized hearing level. Notes: ABR threshold comparisons were performed using the Wilcoxon signed-rank test. Wave V detectability at 40 dB nHL was compared using McNemar’s test for paired binary outcomes.

**Table 3:** Data analysis of the cohorts in terms of wave V latency, amplitude, and statistical significance.

Group	N (Ears)	Latency 60 dB Click	Latency 60 dB CE-Chirp	*p*	Latency 80 dB Click	Latency 80 dB CE-Chirp	*p*	Ln Amp 60 dB Click	Ln Amp 60 dB CE-Chirp	*p*	Ln Amp 80 dB Click	Ln Amp 80 dB CE-Chirp	*p*
Adults Ski-slope HL (overall)	21	6.41 ± 0.58	6.05 ± 0.47	<0.001	5.75 ± 0.40	5.52 ± 0.35	<0.001	−1.81 ± 0.99	−1.60 ± 0.58	0.333	−1.35 ± 0.46	−1.36 ± 0.47	0.489
Adults DROP 1 kHz	9	6.58 ± 0.52	6.11 ± 0.52	0.004	5.93 ± 0.37	5.61 ± 0.35	0.008	−2.29 ± 0.38	−1.95 ± 0.64	0.317	−1.33 ± 0.38	−1.45 ± 0.39	0.312
Adults DROP 2 kHz	12	6.29 ± 0.59	6.00 ± 0.44	0.002	5.61 ± 0.38	5.45 ± 0.35	0.002	−1.67 ± 1.08	−1.47 ± 0.54	0.496	−1.36 ± 0.52	−1.29 ± 0.54	0.910
Children Hearing-impaired	7	6.63 ± 0.44	6.29 ± 0.32	0.016	5.92 ± 0.36	5.69 ± 0.34	0.016	−2.00 ± 0.23	−1.59 ± 0.28	0.031	−1.66 ± 0.43	−1.61 ± 0.37	0.312

Abbreviations: HL, hearing loss; ln, natural logarithm; SD, standard deviation. Notes: ln amplitude refers to the natural log-transformed wave V amplitude values. Within-group comparisons were performed using the Wilcoxon signed-rank test. Latency and amplitude values are expressed as mean ± SD; latency is measured in milliseconds, while amplitude is in microvolts.

## Data Availability

The original contributions presented in this study are included in the article. Further inquiries can be directed to the corresponding authors.

## Abbreviations

The following abbreviations are used in this manuscript:

ABR	Auditory Brainstem Response
IMT	Italian Matrix Test
DPOAE	Distortion Product Otoacoustic Emissions
RNL	Recording Noise Level
NHL	Normalized Hearing Level
NH	Normal Hearing
SD	Standard Deviation
HI	Hearing Impaired
PTA	Pure Tone Average

## References

[1] Maloff E.S., Hood L.J. (2014). A Comparison of Auditory Brain Stem Responses Elicited by Click and Chirp Stimuli in Adults with Normal Hearing and Sensory Hearing Loss. Ear Hear..

[2] Bauch C.D., Olsen W.O. (1988). Auditory Brainstem Responses as a Function of Average Hearing Sensitivity for 2000–4000 Hz. Audiology.

[3] Dau T., Wegner O., Mellert V., Kollmeier B. (2000). Auditory Brainstem Responses with Optimized Chirp Signals Compensating Basilar-Membrane Dispersion. J. Acoust. Soc. Am..

[4] Junius D., Dau T. (2005). Influence of Cochlear Traveling Wave and Neural Adaptation on Auditory Brainstem Responses. Hear. Res..

[5] Chertoff M., Lichtenhan J., Willis M. (2010). Click- and Chirp-Evoked Human Compound Action Potentials. J. Acoust. Soc. Am..

[6] Elberling C., Don M. (2010). A Direct Approach for the Design of Chirp Stimuli Used for the Recording of Auditory Brainstem Responses. J. Acoust. Soc. Am..

[7] Elberling C., Don M., Cebulla M., Stürzebecher E. (2007). Auditory Steady-State Responses to Chirp Stimuli Based on Cochlear Traveling Wave Delay. J. Acoust. Soc. Am..

[8] Wegner O., Dau T. (2002). Frequency Specificity of Chirp-Evoked Auditory Brainstem Responses. J. Acoust. Soc. Am..

[9] Cobb K.M., Stuart A. (2016). Auditory Brainstem Response Thresholds to Air- and Bone-Conducted CE-Chirps in Neonates and Adults. J. Speech Lang. Hear. Res..

[10] Ormundo D.d.S., Fávero M.L., Lewis D.R. (2024). Audiogram Estimation by Auditory Brainstem Response with NB CE-Chirp LS Stimulus in Normal Hearing Infants. Int. Arch. Otorhinolaryngol..

[11] Cho S.-W., Han K.-H., Jang H.-K., Chang S.O., Jung H., Lee J.H. (2015). Auditory Brainstem Responses to CE-Chirp^®^ Stimuli for Normal Ears and Those with Sensorineural Hearing Loss. Int. J. Audiol..

[12] Carhart R. (1945). An Improved Method for Classifying Audiograms. Laryngoscope.

[13] Clark J.G. (1981). Uses and Abuses of Hearing Loss Classification. ASHA.

[14] Garin P., Genard F., Galle C., Jamart J. (2004). The RetroX Auditory Implant for High-Frequency Hearing Loss. Otol. Neurotol..

[15] Pradhananga R.B. (2017). Ski-Slope Hearing Loss and Hybrid Cochlear Implant. Nepal. J. ENT Head Neck Surg..

[16] Schuurbiers J., Dingemanse G., Metselaar M. (2017). Decline of Low-Frequency Hearing in People With Ski-Slope Hearing Loss; Implications for Electrode Array Insertion. Otol. Neurotol..

[17] Moeller M.P. (2000). Early Intervention and Language Development in Children Who Are Deaf and Hard of Hearing. Pediatrics.

[18] Yoshinaga-Itano C., Sedey A.L., Coulter D.K., Mehl A.L. (1998). Language of Early- and Later-Identified Children with Hearing Loss. Pediatrics.

[19] Møller K., Blegvad B. (1976). Brain Stem Responses in Patients with Sensorineural Hearing Loss Monaural Versus Binaural Stimulation. The Significance of the Audiogram Configuration. Scand. Audiol..

[20] Studebaker G.A. (1967). Clinical Masking of the Nontest Ear. J. Speech Hear. Disord..

[21] Norrix L.W., Velenovsky D. (2018). Clinicians’ Guide to Obtaining a Valid Auditory Brainstem Response to Determine Hearing Status: Signal, Noise, and Cross-Checks. Am. J. Audiol..

[22] Foster M., Lightfoot G. (2019). Recommended Procedure: Auditory Brainstem Response (ABR) Testing for Post-Newborn and Adult.

[23] Belet U., Akşit A.M., Kösemihal E. (2025). Comparison of LS CE-Chirp and Click Stimuli in Auditory Brainstem Responses in High-Frequency Hearing Loss. Ear Hear..

[24] Cebulla M., Lurz H., Shehata-Dieler W. (2014). Evaluation of Waveform, Latency and Amplitude Values of Chirp ABR in Newborns. Int. J. Pediatr. Otorhinolaryngol..

[25] Elberling C., Callø J., Don M. (2010). Evaluating Auditory Brainstem Responses to Different Chirp Stimuli at Three Levels of Stimulation. J. Acoust. Soc. Am..

[26] Ormundo D.d.S., Lewis D.R. (2021). Auditory Brainstem Response with Click and CE-Chirp^®^ Level Specific Stimuli in Hearing Infants. Int. J. Pediatr. Otorhinolaryngol..

[27] Mühler R., Rahne T., Verhey J.L. (2013). Auditory Brainstem Responses to Broad-Band Chirps: Amplitude Growth Functions in Sedated and Anaesthetised Infants. Int. J. Pediatr. Otorhinolaryngol..

[28] Stuart A., Cobb K.M. (2014). Effect of Stimulus and Number of Sweeps on the Neonate Auditory Brainstem Response. Ear Hear..

[29] Sininger Y.S., Abdala C., Cone-Wesson B. (1997). Auditory Threshold Sensitivity of the Human Neonate as Measured by the Auditory Brainstem Response. Hear. Res..

[30] Jiang Z.D., Zhang L., Wu Y.Y., Liu X.Y. (1993). Brainstem Auditory Evoked Responses from Birth to Adulthood: Development of Wave Amplitude. Hear. Res..

